# Combined Analytical Study on Chemical Transformations and Detoxification of Model Phenolic Pollutants during Various Advanced Oxidation Treatment Processes

**DOI:** 10.3390/molecules27061935

**Published:** 2022-03-16

**Authors:** Aleksander Kravos, Andreja Žgajnar Gotvajn, Urška Lavrenčič Štangar, Borislav N. Malinović, Helena Prosen

**Affiliations:** 1 University of Ljubljana, Faculty of Chemistry and Chemical Technology, 1000 Ljubljana, Slovenia; aleksander.kravos@fkkt.uni-lj.si (A.K.); andreja.zgajnar@fkkt.uni-lj.si (A.Ž.G.); urska.lavrencic.stangar@fkkt.uni-lj.si (U.L.Š.); 2 University of Banja Luka, Faculty of Technology, 78000 Banja Luka, Bosnia and Herzegovina; borislav.malinovic@tf.unibl.org

**Keywords:** chlorophenols, *Daphnia magna*, electrooxidation, ozonation, phenol, photocatalysis

## Abstract

Advanced oxidation processes (AOPs) have been introduced to deal with different types of water pollution. They cause effective chemical destruction of pollutants, yet leading to a mixture of transformation by-products, rather than complete mineralization. Therefore, the aim of our study was to understand complex degradation processes induced by different AOPs from chemical and ecotoxicological point of view. Phenol, 2,4-dichlorophenol, and pentachlorophenol were used as model pollutants since they are still common industrial chemicals and thus encountered in the aquatic environment. A comprehensive study of efficiency of several AOPs was undertaken by using instrumental analyses along with ecotoxicological assessment. Four approaches were compared: ozonation, photocatalytic oxidation with immobilized nitrogen-doped TiO_2_ thin films, the sequence of both, as well as electrooxidation on boron-doped diamond (BDD) and mixed metal oxide (MMO) anodes. The monitored parameters were: removal of target phenols, dechlorination, transformation products, and ecotoxicological impact. Therefore, HPLC–DAD, GC–MS, UHPLC–MS/MS, ion chromatography, and 48 h inhibition tests on *Daphnia magna* were applied. In addition, pH and total organic carbon (TOC) were measured. Results show that ozonation provides by far the most suitable pattern of degradation accompanied by rapid detoxification. In contrast, photocatalysis was found to be slow and mild, marked by the accumulation of aromatic products. Preozonation reinforces the photocatalytic process. Regarding the electrooxidations, BDD is more effective than MMO, while the degradation pattern and transformation products formed depend on supporting electrolyte.

## 1. Introduction

Today’s highly chemicalized world is far from reaching toxic-free environment. For example, in 2021 European Environment Agency reported that alarming share of European freshwaters during 2013–2019 had excessive levels of pesticides [1]. In a broader sense, less than 38% of waters are claimed to have good status and 75–96% of European seas exhibit contamination issues [2]. The latter points to a fact that no balance between anthropogenic pressure and waters’ self-cleaning capabilities is yet established, even though more than 90% of urban wastewater across the EU is thought to be collected and treated [3]. Therefore, there is a growing commitment to understand and manage pollution, especially with persistent organic micropollutants.

Phenol and chlorophenols (CPs) are representative examples of a wider group of phenolic pollutants. Their presence in the environment is due to intensive historical use, drinking water chlorination, biodegradation of organochlorinated chemicals, and their importance in the chemical industry [4,5,6,7]. Phenol and some CPs are hyper-volume production chemicals, according to OECD. What is more, pentachlorophenol is believed to be the most common chlorinated industrial chemical in the EU [8]. In general, high acute toxicity and genotoxicity are reported, especially for polychlorinated CPs and their degradation products. Nevertheless, total global production of commercial CPs is estimated to tens of kilotons per year and phenol’s production is only slightly less [9]. Used in industry, phenols are prevalent components of industrial wastewaters. Thus, they are continuously transferred into ecosystems and they accumulate in the sediments, as well as biota, where they appear to be ubiquitous. CPs in surface waters reach 2–2000 ng/L [5]; phenols, on the other hand, yet higher concentrations. 

Advanced oxidation processes (AOPs) become well-established technology for water treatment in the last decades. Phenol concentrations > 5 mg/L [10] or even considerably smaller concentrations of CPs that are found in wastewaters are, in practice, biologically non-degradable, but their removal has been proven to be quickly achieved by many physical methods [4], wet-oxidations, ozonation, and many homo-/heterogenic AOPs so far, which include additions of catalysts and/or electro-, photo- or sonochemical treatment [6,7,11,12,13,14]. Considerable research has been taking place also regarding removal of other phenolic pollutants, e.g., nitrophenols, by AOPs that use sustainable materials [6,15]. Nevertheless, despite being optimized and highly effective for the removal of target phenols, complete mineralization is usually not readily achieved by most of the AOPs. Therefore, their chemical pathways from removal of parent compound to the mineralization in connection with the assessment of biological effects remain only rarely studied. For example, the latter aspect has so far been in the case of CPs reviewed by Karci, focusing on Fenton oxidation and UV/H_2_O_2_ treatment [6]. 

Ozonation (OZ) is a ‘quasi’ AOP that has one of the longest histories of use and research, reaching far back in the 20th century. A considerable amount of work has been reported so far on the removal of phenol (PHN) [16,17,18,19,20], 2,4-dichlorophenol (DCP) [18,21,22] and pentachlorophenol (PCP) [22,23,24,25], some reviewed, for example, by Pera-Titus et al. [12] Studies in majority concluded that OZ exhibits high effectivity in removal of target phenol and CPs by progressive formation of multiple C–O and C=O bonds before or after destruction of aromatic ring, as well as cleavage of labile C–Cl, C–H, and C–C bonds. This is possible through molecular or radical mechanism [14]. Yet only the minority of studies on OZ assess ecotoxicity specifically on water flea *Daphnia magna* [26] or study transformation products (TPs) by wide variety of analytical techniques [18,19,20,21,23], especially by mass spectrometry. One important study on degradation pathways—with a wide repertoire of identified TPs—was reported and discussed by Oputu et al. [20] A variety of TPs have been identified so far; the most significant and abundant already in the previous two decades [18,22,27,28].

The synthesis of advanced materials is a driving force for the development of new, increasingly more effective AOPs. Photocatalysis (PC) stands out as a perspective technology. A wide variety of photocatalysts are being assessed, but TiO_2_-based are by far the most prevailing. Use of immobilized (less researched) TiO_2_ thin films on various supports, e.g., glass, metal oxides, and fibres, represents a new alternative to the use of conventional powder forms. Photocatalysts can be, moreover, easily doped with, e.g., Pt, Sb, N, C, thus reinforcing photoactivity [11,29]. Focusing on immobilized TiO_2_, the majority of research is placed on kinetics and target removal of PHN with optimizations of process parameters [30,31], such as those reported by Nickheslat et al. [32], Dougna et al. [33] or Sampaio et al. [34] According to our knowledge, only a minority of studies focuses on DCP [35,36] or PCP removal [36,37]. Moreover, assessments of ecotoxicity or induced chemical transformations are absent; only basic TPs (e.g., hydroxyphenols, organic acids) were identified solely by HPLC–UV [11,31,33,37].

During OZ of phenols, highly oxidized and hydrophilic ring-opening products, such as simple carboxylic acids, are selectively formed but accumulated. Their degradation could be faster by subsequent PC. Such complementarity has been stimulating interest in the sequential method (SQ) [38,39], but research on it is scarce, according to our survey. 

AOPs are continuously being developed to reach a ‘low-cost, high-tech, chemical-free’ ideal. One such opportunity has been seen in electrochemical oxidation (EO), by which degradation is achieved at mild conditions during electrolysis [7,13]. Lab-scale optimizations of parameters—using boron-doped diamond [40,41,42] or metal-oxide [41,42,43,44] anodes in different supporting electrolytes—to reach optimal phenol removal efficiency versus energy consumption are being widely reported. Meanwhile, monitoring the evolution and toxicity of formed TPs is highly disregarded. Significant research on this matter (PHN only) has been, for example, performed by Jiang et al. [40], Barışçı et al. [44], Amado-Piña et al. [17], and Xing et al. [41], again mainly using HPLC–UV. 

There is a lack of knowledge of induced degradation processes and pathways, for which there are no in-depth studies and comparisons available. As so, we focused on profound analyses of degradation processes of PHN, DCP and PCP induced by: OZ; PC with *N*-doped TiO_2_ thin films on glass plates (*N*-TiO_2_*^im.^*) and photooxidation (PO); SQ (OZ followed by PC); and EO either with boron-doped diamond (BDD) or mixed-metal oxide RuO_2_-IrO_2_ (MMO) anode in two different supporting electrolytes. A wide range of analytical techniques were applied to obtain information about parent phenol removal, degradation progressivity, mineralization, and dechlorination, along with evolution of TPs. Moreover, ecotoxicological insight was gained by assessing ecotoxicity of treated fractions on water flea *Daphnia magna* for the first time for some of the above AOPs.

## 2. Materials and Methods

### 2.1. Chemicals and AOP Treatment

*Chemicals.* All the chemicals in the present study were bought from Sigma-Aldrich (Steinheim, Germany), Fluka (Seelze, Switzerland), Merck (Darmstadt, Germany), Kemika (Zagreb, Croatia), Fisher Scientific (Loughborough, UK), etc. Purity was at least *p.a.* or was not less than 98%. Further details about standards, reagents, solvents, materials for AOPs, and additional chemicals for identification can be found in Supplementary Materials (Section S1).

*General AOP treatment procedure.* Prepared test mixture (Table 1) was transferred into a reactor and treated with the selected method. During treatment, sampling was performed at exact time intervals. Collected aliquots (2–20 mL) were stored in plastic vials in freezer at −20 °C. Detailed descriptions of procedures are accessible in the Supplementary Materials (Sections S2–S5). PCP solutions were always prepared in concentrations less or equal to approximately 10 mg/L due to poor solubility in water.

*AOP materials & configurations.* During ozonation, the gaseous mixture O_2_/O_3_ was continuously introduced in the reactor containing test solutions. Photocatalysis was achieved by using *N-*doped TiO_2_ synthesized by the sol-gel method from a TiCl_4_ precursor. It was immobilized on glass plates in the form of thin films using the dip-coating technique. A photocell with a continuous flow of O_2_ placed in a UVA-illuminator was used. Electrooxidation was achieved in an electrochemical cell with a mesh-type anode (BDD, MMO) and cathode (stainless steel). For details see the Supplementary Materials (Sections S2–S5).

### 2.2. Instrumental Analysis

*High-performance liquid chromatography coupled to diode-array UV detection.* HPLC–DAD (HPLC System 1100 Series, Agilent Technologies, Santa Clara, CA, USA) was used for determination of target phenols (PHN, DCP, PCP) and chosen TPs (hydroquinone, catechol, tetrachlorohydroquinone), semiquantitative estimation of *p-*benzoquinone, and a number of chromatographic peaks (with UV-absorptivity). The used column was Kinetex XB-C18, 150 mm × 4.6 mm, 5 µm, 100 Å (Phenomenex, Torrance, CA, USA) and guard column Gemini-C18, (Phenomenex, Torrance, CA, USA). Flow rate was 0.7 mL/min. Conditions I were: mobile phase A—10% acetic acid in MQ; B—acetonitrile; separation programme (min-%A/%B): 0–90/10, 3–90/10, 10–60/40, 17–20/80, 23–20/80, 24–90/10, 25–90/10, 2 min post time; UV-Vis detection at 254, 270, 285, 305 nm. Conditions II were: mobile phase A—10 mM H_3_PO_4_ in MQ; B—acetonitrile; separation programme (min-%A/%B): 0–100/0, 6–100/0, 9–40/60, 12–20/80, 15–20/80, 16–100/0, 17–100/0, 2 min post time; UV-Vis detection at 254 and 210 nm. 

*Ultra-high-pressure liquid chromatography coupled to mass spectrometry.* Specific TPs were tracked by LC–MS/MS (Vanquish LC System, TSQ Quantis, Thermo Fisher Scientific, Waltham, MA, USA) with negative electrospray ionization. The column, guard column and flow rate were the same as in HPLC–DAD. Mobile phase composition was A—0.1% formic acid in MQ and B—acetonitrile; separation programme (min-%A/%B): 0–90/10, 3–90/10, 10–60/40, 17–20/80, 23–20/80, 24–90/10, 25–90/10, 2 min post time. For MS analysis, N_2_ (Messer, Bad Soden, Germany, 99.999%) was used as sheath/aux/sweep gas (AU): 70/24/0.5. The ion source was at 385 °C and the nebulizer gas was at 520 °C. Capillary voltage was set to −200 V. Spectra were recorded in TIC full Q3 scan mode (*m/z* 61–355) with no source fragmentation nor collision-induced dissociations in collision cell. 

*Gas chromatography coupled to mass spectrometry.* Volatile and semipolar products were selectively extracted by solid-phase microextraction (SPME) by immersion of the CAR-PDMS, PA or PEG fibre (Supelco, Bellefonte, PA, USA) for 30 min at 30 °C in a 5 mL sample with 0.2 mL of 0.1 M H_2_SO_4_ added. Liquid–liquid extraction (LLE) in ethyl acetate and *n-*hexane was also conducted. Either LLE or SPME extracts were analysed by GC–MS (FOCUS GC, ISQ, Thermo Fisher Scientific, Waltham, MA, USA). Conditions I were: DB-624 column (30 m × 0.25 mm, 1 μm, Agilent J&W, Folsom, CA, USA); He flow was set to 0.8 mL/min; splitless injection with surge mode; inlet temperature 260 °C; temperature programme (50 °C, 5 min; 110 °C, 10 °C/min; 210 °C, 15 °C/min, 3 min; 230 °C, 10 °C/min, 8 min; 240 °C, 10 °C/min, 10 min; 250 °C, 10 °C/min); ion source temperature 250 °C; MS transfer temperature 250 °C; MS was operated in TIC mode in the range *m/z* 42–350. LLE extracts were also analysed with GC–MS/MS (TRACE 1300 GC, TSQ 9000, Thermo Fisher Scientific, Waltham, MA, USA) at the following conditions II: HP-5MS column (30 m × 0.25 mm/0.25 μm, Agilent J&W, Folsom, CA, USA); He flow set to 1.0 mL/min; splitless injection; inlet temperature 280 °C; temperature programme (50 °C, 3 min; 60 °C, 2 °C/min, 1 min; 140 °C, 5 °C/min, 1 min; 320 °C, 10 °C/min, 1 min); ion source temperature 280 °C; MS transfer temperature 280 °C; MS was operated in TIC mode in the range *m/z* 42–350. 

*Ion chromatography.* Protic species were identified (succinate/malate) and quantified (formiate, oxalate/fumarate, maleate, and Cl^−^) or semiquantified (glyoxylate/glycolate/acetate) by anion-exchange IC (Dionex ICS 5000, Thermo Scientific, Sunnyvale, CA, USA), consisting of a gradient pump, an electrochemical suppressor (Dionex AERS 500, 4 mm) and a conductivity detector. The column was AS11-HC (4 × 250 mm, Dionex, Thermo Scientific, Sunnyvale, CA, USA) and flow rate 1.0 mL/min. Mobile phase composition was A—MQ, B—10 mM NaOH in MQ, C—100 mM NaOH in MQ. Separation programme (time-A/B/C): 0–95/5/0, 30–85/15/0, 35–70/15/15, 55–67/15/18, 60–60/15/25, 70–95/5/0, 10 min post time). Suppressor was set to 50 mA.

*Total organic carbon (TOC), pH measurements, and UV spectroscopy.* TOC (TOC multi N/C 3100, Analytik Jena, Jena, Germany) according to ISO 8245, 1999, pH (SevenEasy, Mettler Toledo, Columbus, OH, USA) and UV spectra (Agilent Cary 60 UV-Vis, Agilent Technologies, Santa Clara, CA, USA) in the range 200–450 nm were additionally determined in some cases for non-specific estimation of mineralization, evolution of acids or changes in chromophores. 

### 2.3. Inhibition on Daphnia magna

Ecotoxicity was assessed by 48 h ecotoxicological testing of acute inhibition of mobility of water flea *Daphnia magna* (Cladocera, Crustacea) according to standard protocol described in OECD Guidelines No. 202 [45]. More details are given in the Supplementary Materials (Section S6).

Each treated sample (i.e., ozonated, photocatalyzed, photooxidized, electrooxidized) was previously diluted with OECD test medium (test mixture) by appropriate factor to reach the referential ‘test’ concentration of target phenol (*γ_x_*). Chosen *γ_x_* for PHN (50 mg/L), PHN (10 mg/L), PHN for EO by BDD/NaCl, DCP, and PCP were 10.0, 5.0, 5.0, 2.5, 0.6 mg/L, respectively. For example, ozonated 10 or 50 mg/L DCP sample at a chosen treatment time was diluted 4 or 20 times, respectively. The tests were conducted in 3 separate determinations in microtiter plates, each containing 10 mL of chosen test mixture and 10 less than 24 h old *Daphnia* offspring were added. Incubation of organisms in samples was conducted for 48 h in the dark at room temperature. After the incubation, inhibition (%*_inh_*) was determined according to Equation (4) in Section 2.4. 

### 2.4. Numerical Evaluation

Approximate treatment time needed for reaching *x*% removal of target phenol (TT*_x_*_%_).

Approximation of the estimated pseudo 1st kinetic order constant of degradation (*k*_r_)

(1) $$k_{r}=\frac{ln\left(2\right)}{estimated\;half\;time\;\left(t_{1/2}\right)}\;\mathrm{OR}\;\mathrm{estimated}\;\mathrm{from}\;\mathrm{graphs}\;ln\left[\mathrm{parent}\;\mathrm{phenol}\right]=f\left(\mathrm{TT}\right)$$

Approximate level of mineralization at a certain time (%*_min_*)

(2) $${\%}_{min}=100-100\times \frac{TOC\;\left(\mathrm{treated}\;\mathrm{sample}\right)}{TOC\;\left(\mathrm{untreated}\;\mathrm{sample}\right)}$$

Dechlorination extent at a certain treatment time (%*_dec_*)

(3) $$\begin{array}{c} {\%}_{dec}=\frac{moles\;\left({\mathrm{Cl}}^{-}\;\mathrm{in}\;\mathrm{treated}\;\mathrm{sample}\right)-moles\;\left({\mathrm{Cl}}^{-}\mathrm{in}\;\mathrm{untreated}\;\mathrm{sample}\right)}{y\times moles\;\left(\mathrm{DCP}\;\mathrm{and}/or\;\mathrm{PCP}\;\mathrm{in}\;\mathrm{untreated}\;\mathrm{sample}\right)}\times 100\\ y=2\;\mathrm{for}\;\mathrm{DCP};\;y=5\;\mathrm{for}\;\mathrm{PCP} \end{array}$$

Normalized relative amount of chosen transformation product (< product >)

(4) $$<\mathrm{product}>\;=\frac{peak\;area\;in\;chromatogram\;\left(\mathrm{chosen}\;\mathrm{product}\right)}{the\;biggest\;peak\;area\;\left(\mathrm{chosen}\;\mathrm{product}\;\mathrm{in}\;\mathrm{the}\;\mathrm{same}\;\mathrm{set}\;\mathrm{of}\;\mathrm{samples}\right)}$$

Relative descriptor of a chosen transformation product (RD)

(5) $$\begin{array}{c} {\mathrm{RD}}_{prod.}=\frac{\gamma \;\left(\mathrm{product}\;\mathrm{in}\;\mathrm{treated}\;\mathrm{sample}\right)\times molar\;mass\;\left(\mathrm{parent}\;\mathrm{phenol}\right)}{E_{ff}\times a\times \gamma \;\left(\mathrm{parent}\;\mathrm{phenol}\;\mathrm{in}\;\mathrm{untreated}\;\mathrm{sample}\right)\times molar\;mass\;\left(\mathrm{product}\right)}\\ \left(E_{ff}=\frac{\gamma \;\left(\mathrm{parent}\;\mathrm{phenol}\;\mathrm{in}\;\mathrm{treated}\;\mathrm{sample}\right)\;}{\gamma \;\left(\mathrm{parent}\;\mathrm{phenol}\;\mathrm{in}\;\mathrm{untreated}\;\mathrm{sample}\right)}\right) \end{array}$$

Complete theoretical conversion ‘C_6_H_5–*x*_OCl_*x* → *a*_ chosen product’ (*a* is 3 and 6 for oxalic and formic acid, respectively) is assumed, regardless of other chemical transformations and changes in the volume of test mixture due to sampling during AOP treatment.

Acute 48-h mobility inhibition with water flea *Daphnia magna* (%*_inh_*)

(6) $${\%}_{inh}=\frac{No.\;\mathrm{of}\;\mathrm{immobilized}\;Daphnia\;\mathrm{after}\;\mathrm{incubation}\;}{No.\;\mathrm{of}\;Daphnia\;\mathrm{in}\;\mathrm{the}\;\mathrm{test}\;\mathrm{mixture}\;\mathrm{at}\;\mathrm{the}\;\mathrm{beginning}\;}\times 100$$

## 3. Results and Discussion

The motivation was to fully understand complex degradation processes of phenol (PHN), 2,4-dichlorophenol (DCP), and pentachlorophenol (PCP) in different matrices from chemical and ecotoxicological point of view. A focus was placed on profound analyses of treated fractions. This allowed a comparison of four approaches for chemical degradation: ozonation (OZ), photocatalytic oxidation with immobilized *N*-doped TiO_2_ thin films on glass supports (PC), their sequence (SQ), and anodic electrooxidations (EO) by BDD and MMO anode, thus covering a wide range of three advanced technologies.

In order to collect data on target degradation progress, dechlorination, mineralization, changes in pH, chemical transformations, and evolution of selected by-products, numerous analytical methods and procedures were applied. Such as: HPLC–DAD, pH measurement, TOC determinations, UV spectroscopy, solid-phase microextraction (SPME) or liquid–liquid extraction (LLE) followed by GC–MS or GC–MS/MS, UHPLC–MS/MS, ion chromatography (IC), and ecotoxicological mobility inhibition tests on *Daphnia magna* water flea. All of these are further described in the following section.

### 3.1. Removal of Target Phenols, Mineralization, and Progressivity

To describe the efficiency of the removal, we used several descriptors that are explained in Section 2.4, namely: treatment time (TT) needed for reaching > 95% target phenol removal (TT_>95%_), estimated pseudo first-order kinetics constant (*k*_r_; Equation (1)), and level of mineralization (%*_min_*; Equation (2)).

**Figure 1:**
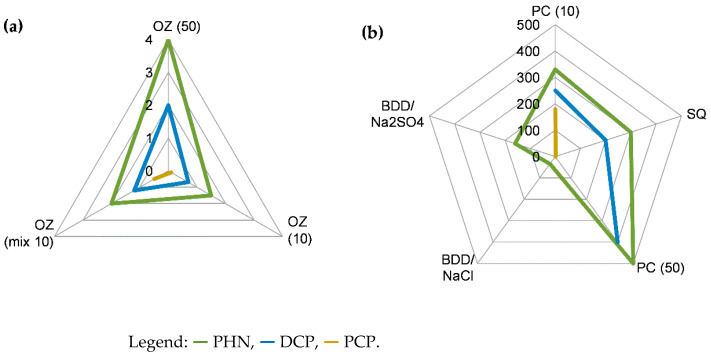
Treatment time for reaching > 95% removal (see Section 2.4) of phenol (PHN), 2,4-dichlorophenol (DCP) and pentachlorophenol (PCP) (TT_>95%_ in min) after treatment with (**a**) ozonation (OZ), and (**b**) photocatalysis (PC), sequential method (SQ), and electrooxidation with BDD anode in NaCl (BDD/NaCl) and Na_2_SO_4_ (BDD/Na_2_SO_4_) (note: numbers in parentheses are initial concentrations in mg/L).

*Ozonation (OZ).* Results indicate that at the initial pH of 8, a favourable degradation process of phenols is possible, which includes rapid target degradation, depending on their chemical structure (correlation with nucleophilicity, mechanism, and intermediates), medium complexity (competition for O_3_ consumption), and initial concentrations (substrate loads). All of that is reflected in Figure 1a and is in agreement with the literature [12,18]. In all cases, pseudo first-order kinetics could be approximated, e.g., estimated pseudo first-order constants (*k*_r_) for PHN and DCP in separate solutions reached 0.6 and 2.1 min^−1^ (50 mg/L), respectively, whereas for the mixture they were 1.9, 2.8, and 4.9 min^−1^ for PHN, DCP, and PCP, respectively. The approximate TT_>95%_ were 4, 2, and ~0.1 min for PHN, DCP (50 mg/L), and PCP (10 mg/L), respectively (Figure 1a). The higher the initial concentration or the greater the amount of co-substrates (see OZ of mixture), the longer TT_>95%_. Described progressivity was primarily reflected in: (i) a sudden drop of pH from 8 to 3–4, with the formation of organic acids; (ii) compounds detected by HPLC–DAD (e.g., absorption decline < 255 nm, as well as evolution of extra polar products with minimum retention); and (iii) an increase in the number of IC peaks (showing a quick evolution of protic species), presented in the Supplementary Materials (Section S2.1). Since OZ proceeds in acidic medium, reactions of substrates with O_3_ were assumed to be taking part on the gas–liquid interface, especially in the early stage [24,27]. Reaching strongly acidic pH was thought to greatly influence degradation process. Mineralization was, on the other hand, not readily achieved. For example, only 50, 40, and 40%*_min_* for PHN, DCP, and PCP (10 mg/L), respectively, was measured after 10 min.

**Figure 2:**
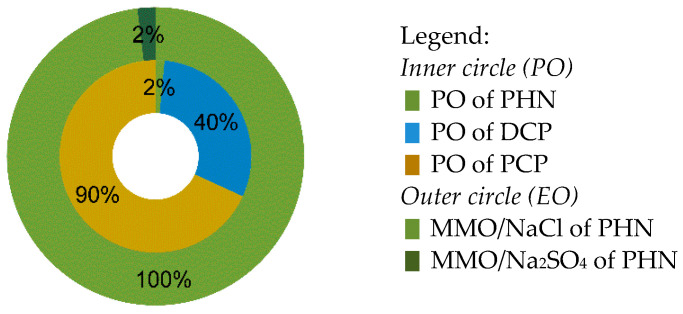
Maximum removal % of phenol (PHN), 2,4-dichlorophenol (DCP), and pentachlorophenol (PCP) achieved by 300 min of photooxidation (PO), 120 min of electrooxidation with MMO in NaCl, and 160 min with MMO in Na_2_SO_4_.

*Photocatalysis (PC) and photooxidation (PO).* In general, selective, robust, and rapid degradation is considered for OZ, while mild and slow degradation is known for PC. Data concerning PC (i.e., UV/O_2_/TiO_2_*^im.^* system) indicate up to a 100-times longer treatment time (reaching few hours) than those characteristic for OZ (reaching few minutes). TT also depends on specific parameters, such as number of C–Cl bonds [35,36] and initial concentration [35]. This is in accordance with [12,27] which reported that PC is significantly less efficient than OZ. For example, estimated *k*_r_ was 0.01, 0.02, and 0.03 min^−1^ for PHN, DCP, and PCP (10 mg/L), respectively, but 0.005 and 0.008 min^−1^ for PHN and DCP (50 mg/L), respectively. Therefore, TT_>95%_ are above the 3-h time range. By increasing the number of C–Cl bonds and initial concentration, removal was faster (Figure 1b), which shows phenols’ reactivity. PCP has the greatest number of labile C–Cl bonds, the smallest p*K_a_*, and the highest UVA absorptivity (at 365 nm); therefore, its degradation was the fastest. PO (i.e., UV/O_2_ system; absence of *N*-TiO_2_*^im.^*) was found to be the least effective and the slowest, since removal efficiency was up to 500 times lower than for PC. This is especially true in the case of PHN where only 2% removal was reached after 5 h treatment. In addition, estimated *k*_r_ were <10^−5^, <10^−3^, and 0.006 min^−1^ for PHN, DCP (50 mg/L), and PCP (10 mg/L), respectively (Figure 2). Effect of UV light was, therefore, the highest in the case of PCP (90% removal reached after 5 h) due to the progressive breakage of C–Cl bonds on the aromatic ring. Mineralization after >180 min of PC treatment was estimated to be 60, 70, and 60% for PHN, DCP, and PCP (10 mg/L), respectively, whereas no mineralization was induced by PO. UV spectroscopical data on PC of PHN and DCP (50 mg/L) further support the facts described above, as there was a slow decrease in characteristic peak absorptivity (270 nm for PHN, 285 nm for DCP) during treatment. Finally, assessment of HPLC and IC chromatogram peaks may give overall conclusion that PC and PO processes are more selective, and less dynamic in contrast to OZ (see Supplementary Materials Section S3.1).

*Sequential method (SQ).* In the case of SQ, a direct comparison to PC or OZ alone is not possible due to different initial concentrations used in experiments. Nevertheless, experiments show a contrast between OZ and PC, where OZ was considerably faster (Figure 1b), as already discussed. For example, a 0.2 min ‘flash’ of OZ removed almost all PCP and approximately 75% of DCP and 25% of PHN. So, SQ may provide a faster option as opposed to slow PC. Examples of chromatograms are given in the Supplementary Materials (Section S4.1). On the other hand, it should be noted that during OZ, the pH suddenly dropped to <6 while, interestingly, during PC the pH did not change significantly. 

*Electrooxidation (EO).* Only PHN degradation (50 mg/L) was investigated by EO. Removal was the fastest in NaCl as the supporting electrolyte. In the case of BDD/NaCl, TT_>95%_ was only below 35 min (Figure 1b) but chlorinated aromatics were formed which were almost completely removed after 60 min. Whereas in the case of MMO, TT_>95%_ was up to 120 min; also due to mild polychlorination (Figure 2). The process was the most progressive with BDD, which can be estimated also from HPLC chromatograms. On the other hand, EO in Na_2_SO_4_ was slower, selective, and less dynamic. Less HPLC–DAD-detectable peaks were generated in comparison to EO/NaCl (see the Supplementary Materials, Section S5.1). If BDD and MMO are compared, treatment with BDD was effective on a long run, as 96% removal after 160 min was achieved (Figure 1b). In contrast, an MMO anode provided only less than 10% removal (Figure 2). Data are comparable to the literature [17,40,44].

### 3.2. Dechlorination of Chlorophenols

Monitoring of Cl^−^ concentration allowed us to track the breakage of C–Cl bonds, referred to as dechlorination. From it, the overall amount of remaining chlorinated compounds can be estimated. The applied descriptor was the dechlorination extent (%*_dec_*), explained in Section 2.4 (Equation (3)).

**Figure 3:**
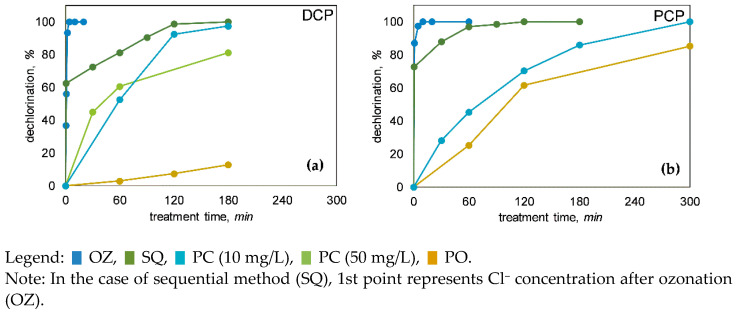
Dechlorination (i.e., conversion of organic chlorine into chloride; see Equation (3) in Section 2.4) of (**a**) 2,4-dichlorophenol (DCP) and (**b**) pentachlorophenol (PCP) during ozonation (OZ), photocatalysis (PC), photooxidation (PO), and sequential method (SQ).

*Ozonation*. During OZ, fast (<3 min) and complete dechlorination could be observed. Curves for dechlorination extent (Figure 3) are in all cases of the same shape, marked by a fast increase in Cl^−^ concentration, but more steady changes in the later stage of OZ. This points to the probability that more labile C–Cl bonds on C atoms that are part of aromatic structures (Ar–Cl) are quickly broken. The same is valid for OZ of mixture, where 100% combined dechlorination of PCP and DCP was reached after only 3–4 min (data not shown). All in all, non-chlorinated TPs were in the majority expected during and after OZ, which is favourable.

*Photocatalysis and photooxidation*. During PC, the dechlorination process was much slower than in OZ, expanding to 2–5 h (Figure 3), which is proportional to slower removal efficiency (see Section 3.1). For example, in the first hour of PC there was still >50% of chlorinated organic compounds (including non-degraded DCP and PCP), and then from the third hour onwards, most of the chlorine was already in the form of Cl^−^, which is favourable (Figure 3). Nevertheless, long-term dechlorination efficiency could be predicted since more than 80%*_dec_* was achieved after a 5-h treatment. In the PO experiments, cleavages of C–Cl also occurred, especially in the case of PCP, rather than DCP (Figure 3b), suggesting that UVA irradiation plays an important role not only in PCP removal but also in its dechlorination (<60%*_dec_* reached after 5 h). In comparison, Gunlazuardi and Lindu [37] reported on the much slower PCP’s release of Cl^−^ ions. 

*Sequential method*. Similar to removal efficiency (Figure 1), OZ with subsequent PC also reinforced dechlorination process (Figure 3). With 0.2 min of OZ, it was possible to declare >60%*_dec_* and fast dechlorination. However, with subsequent PC, the process steadily slowed down.

### 3.3. Transformation Products and Possible Degradation Pathways

#### 3.3.1. General Identification

Several analytical techniques were applied to separate and identify the transformation products (TPs) forming during treatment: HPLC–DAD, UHPLC–MS/MS, liquid–liquid extraction (LLE) or solid-phase microextraction (SPME) followed by GC–MS, LLE followed by GC–MS/MS, and IC (see Section 2.2). A list of some identified TPs is given in Table 2, where they are numbered from A1 to D16. A part of TPs were identified by several analytical techniques, while some of them only by one of the applied techniques, thus lowering the identification certainty. 

**Figure 4:**
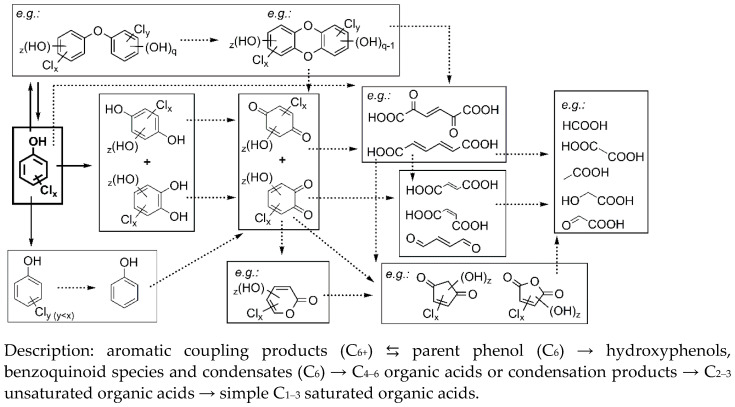
General scheme of the suggested degradation process during ozonation (partially adopted from [6,16,17,18,19,20,21,24,28]).

*Ozonation.* OZ is considered very effective in destruction of the phenols’ stable aromatic structure in contrast to other AOPs due to direct reaction with bipolar O_3_ molecule [14,17,20]. In general, degradation is characterized by many stages, as suggested by Figure 4. The first indicator of any chemical transformation was the change in colour of solutions. Firstly (Table 2), rapid and simultaneous formation of 1,2- and 1,4-aromates, e.g., hydroxyphenols, benzoquinoids (B12,14,15, C1–6), and others (A10–16, B2–6,7) characteristically occurred, reported also in [20,21,23,24], due to substitutions on *orto* and *para* sites with further oxidations [24]. In smaller extent, partially dechlorinated CPs were formed (B10), also mentioned in [18,25]. Opening of the oxidized aromatics’ benzene ring may have led to the formation of multifunctional carbonyl compounds, and from there on to condensation and cyclization reactions leading to furans, cyclopentanones etc. (C9–15), similarly as in [16,17,20,27]. A degradation process is highly progressive, but it was terminated by the formation of C1–4 simple organic acids (e.g., oxalic, formic, acetic; D9,12–16) that are known to accumulate because of their stability towards O_3_ [17,24,28]. Further reactions between acids, oxidations [20], cyclizations, cycloadditions, peroxidations, redox transformations [17], addition reactions with bond breakages [28], and further ozonolysis might proceed as well, as suggested from literature [20]. In addition, the initial stages of OZ and higher initial concentrations of phenols brought about oxidative coupling reactions, resulting in formation of chlorinated and/or polyhydroxylated coupling products in traces (most probably phenoxyphenols, biphenyls, dibenzodioxins and others; A1–5,7). Their *m/z* with isotopic fingerprints were specifically detected by UHPLC–MS/MS (Table 2) due to good ionization by negative ESI and are therefore only suggested as possible, but those TPs were highlighted also by Oputu et al. [20] and Hirvonen et al. [22]. The respective products were more numerous in the case of OZ of CPs. For example, number of coupling products was 1 and 8 for PHN and DCP, respectively. These reactions were also non-specifically confirmed by a sudden drop in pH and by number and peak types detected by HPLC–DAD and IC. The above highlighted and many other transformations are described also in literature [6,16,18,19,20,21,24,28], especially recently by Oputu et al. in 2020 [20]. For example, decrease in absorption < 255 nm indicated a rapid formation of simple acyclic compounds, such as organic acids.

*Photocatalysis and photooxidation*. In the literature, target identifications of TPs in PC are prevailing, using only HPLC–UV. A look into identified TPs (Table 2) suggests that PC was incapable to efficiently open the aromatic ring. Thus, there was a prevalence of reactions on the aromatic ring [27]. For example, degradation was marked by formation of benzoquinones, hydroxyphenols (B10,12,14,15), less chlorinated chlorophenols (B10) [36], as well as dimers, adducts, biphenyls or phenoxyphenols which are thought to be product of (oxidative) coupling, i.e., formations of C–O and C–C bonds (A1–5,7). These predominated in the first 2 h of PC, and mostly in the case of PO, so their formation was accelerated by UVA irradiation. The formation of all mentioned aromatics was a result of radical hydroxylations, photoinduced oxidations, reductive/hydroxylative dechlorinations, and other substitution reactions. Consequently, CPs, hydroxyphenols, and benzoquinones are by far the most frequently identified in the literature, such as hydroquinone (C4), catechol (C6) and *p-*benzoquinone (C2) [11,31,33,37]. UVA irradiation plays a significant role in the cleavage of C–Cl bonds and coupling reactions, but only the presence of photocatalyst allows for increased destruction of aromatic structures. Radical reactions induce also other transformations, such as photooxidations, cyclizations, coupling reactions, and condensations resulting in evolution of simpler compounds, e.g., hydroxycyclopentanediones (C13,14), reported also in [35]. Cyclized organic oxygen derivatives (C11,13,14, D1), and organic acids (D9) also appeared during PC, but in very low concentrations, and are reported also in [11,12]. As dechlorination was slow, more chlorinated aromatics were detected. In the case of PC of PHN, UV spectroscopy showed a gradual increase in the secondary absorption peak ranging 280–310 nm, which could have possibly indicated the formation of various quinoid or condensed aromatic species. During treatment, the overall absorptivity gradually decreased, and the absorption peaks were no longer clearly defined. Moreover, there were less HPLC–DAD peaks, which would indicate formation of simple non-aromatic compounds.

*Sequential method.* TPs in SQ treated samples were similar to those in OZ which were then further degraded by PC (Table 2). Interestingly, there were fewer coupling products detected although they are otherwise typical of PC; possibly because of preozonation.

*Electrooxidation.* As already mentioned, EO in electrolyte NaCl is the most effective for the removal of PHN, yet the least successful since there was unfavourable formation of chlorinated aromatics due to in situ electrogeneration of chlorinating agents. In the case of BDD/NaCl, chlorinated aromatics (mostly chlorophenols, B10, reported also by Chatzisymeo et al. [43]) were preferentially formed (Table 2). However, after 60 min they were broken down into chlorinated carbonyl compounds and polychlorohydrocarbons (C8–10,14,16, D2,3,6–8,10,11), e.g., chloroform, tetra/pentachloropropenes, tetrachloroetene, tetrachlorocyclopropanes, etc. Thus, ring-opening reactions effectively occurred. MMO/NaCl treatment was characterized by an even greater generation of chlorinated aromatics which were accumulated (A1–5,7, B10,12, C3). For example, even after 120 min of EO they still prevailed; contrarily, in the case of BDD they were quickly degraded. Despite extensive and rapid target degradation of PHN in NaCl, we cannot speak of a successful process.

On the other hand, slower EO in Na_2_SO_4_ provided more acceptable chemical transformations. Non-chlorinated, hydroxylated and/or highly oxidized aromatics were preferentially formed (Table 2), such as hydroquinone, catechol, *p-*benzoquinone, and organic acids [40,44], as well as some coupling products. On the long run, aromatic ring might have been opened. MMO was an exception since progressivity was slower. Thus, *p-*benzoquinone accumulated in a relatively big proportion. Moreover, there were fewer TPs identified than during EO/NaCl. In addition, probably relatively greater amount of *p-*benzoquinone was generated by MMO than by BDD, as estimated from peak areas in HPLC–DAD. Hydroquinone, catechol, *p-*benzoquinone, and organic acids (e.g., fumaric, oxalic, maleic) formation were in majority identified by target analysis with HPLC–UV also in the literature [17,40,42,44]. 

#### 3.3.2. Monitoring of the Selected Products 

**Figure 5:**
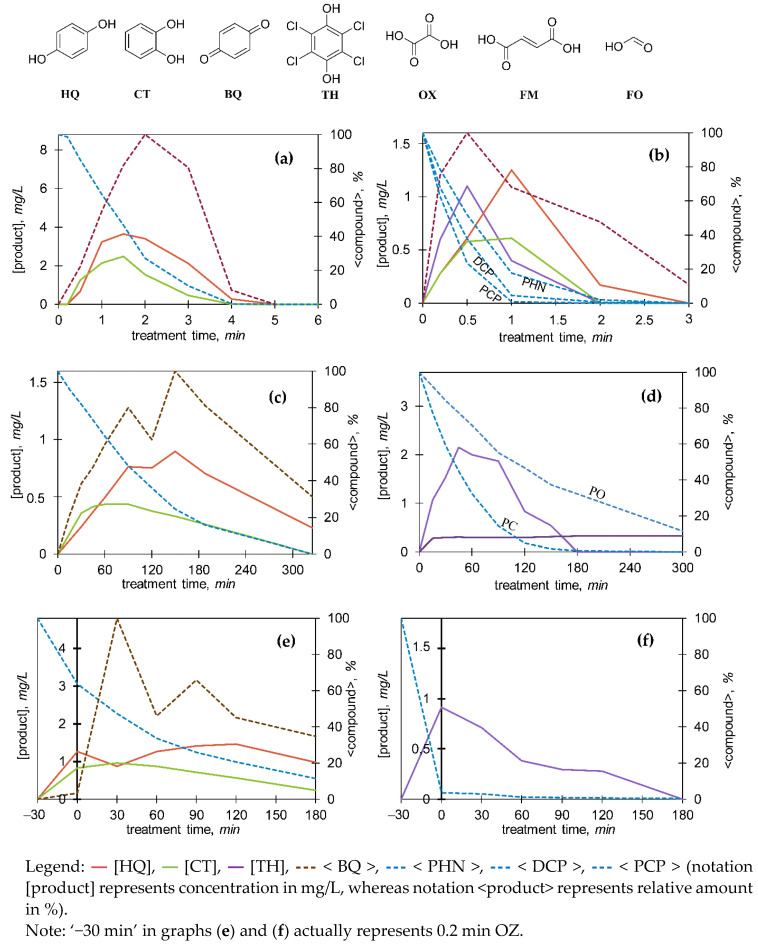
Concentrations (left *y*-axis) of hydroquinone (HQ), catechol (CT), and tetrachlorohydroquinone (TH), and normalized relative amount (right *y*-axis; see Equation (4) in Section 2.4) of parent phenols (PHN, DCP, PCP) and *p*-benzoquinone (BQ) during (**a**) ozonation of phenol (PHN) (50 mg/L); (**b**) ozonation of mixture (10 mg/L each); (**c**) photocatalysis of PHN (10 mg/L); (**d**) photocatalysis and photooxidation of pentachlorophenol (PCP) (10 mg/L); (**e**) sequential method of PHN (approximately 20 mg/L); (**f**) sequential method of PCP (approximately 10 mg/L).

**Figure 6:**
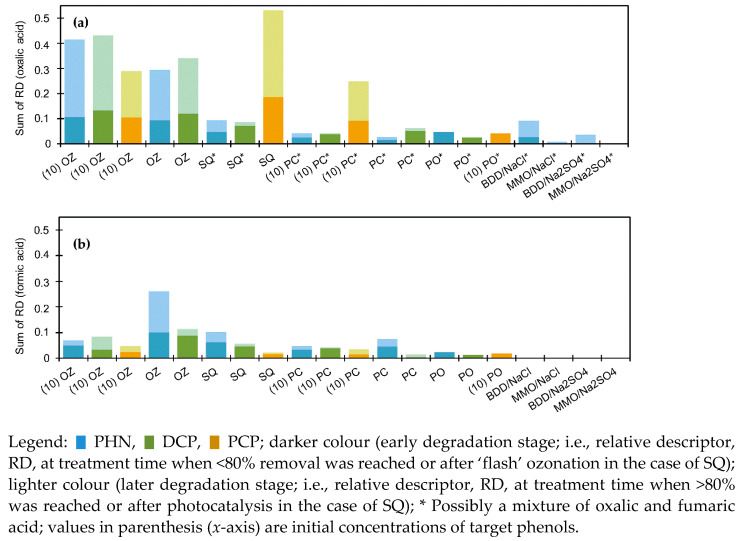
Presence of (**a**) oxalic and (**b**) formic acid in early and later degradation stages (quantified by relative descriptor, RD, according to Equation (5) in Section 2.4) of phenol (PHN), 2,4-dichlorophenol (DCP) and pentachlorophenol (PCP) reached by ozonation (OZ), photocatalysis (PC), photooxidation (PO), and electrooxidation (EO) with BDD and MMO anode in NaCl and Na_2_SO_4_.

Hydroquinone (HQ), catechol (CT), tetrachlorohydroquinone (TH), and organic acids (oxalic, OX; formic acid, FO) were quantified from their respective calibration curves obtained by HPLC–DAD and IC (Figure 5 and Figure 6). By IC, acetic/glyoxylic/glycolic, maleic, succinic/malic, propionic, lactic, fumaric acid (FM; note: coelution with oxalic acid but differentiated by HPLC–DAD) could also be detected. Relative abundance of *p-*benzoquinone (BQ), as well as other identified TPs was monitored according to Equation (4) (Section 2.4).

*Ozonation.* The results generally show that organic acids were rapidly formed during OZ (mostly oxalic, but also formic, maleic, and acetic/glyoxylic/glycolic acid); those were slowly degraded afterwards (Figure 6). Monitoring of aromatic representatives (HQ, CT, TH, BQ; Figure 5a,b) indicate that their formation was favourable only in the initial OZ stages, which means that the aromatic ring was later opened due to ozonolysis, resulting in acyclic compounds and/or their condensates. Interestingly, HQ evolution was more favourable than CT’s (Figure 5a,b).

**Figure 7:**
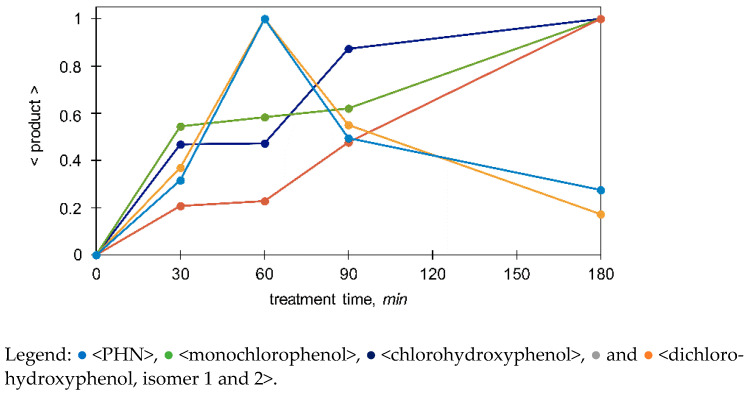
Normalized relative amount of chosen products (see Equation (4) in Section 2.4) in ozonated 2,4-dichlorophenol (DCP) (50 mg/L) determined by SPME/GC–MS.

*Photocatalysis and Photooxidation.* Unlike with OZ, the amounts of HQ, CT, BQ, and TH were considerable, and they were long-lasting. For example, during PC of PHN, concentrations of HQ and CT were reaching up to approximately 7 and 3 mg/L, respectively, while during PO of PHN concentrations were only 0.3 and <0.2 mg/L (data not shown), respectively. Similar examples are given in Figure 5c,d. Thus, the formation of organic acids was much slower (similar to slower degradation process), reaching lower concentration ranges (Figure 6). CT formation was, again, slower than HQ’s and it reached lower concentrations in both PC and PO processes, reported also in [34]. In the case of PCP degradation, formation of quite persistent TH stands out, indicating substitution reaction on the *para* site. UVA irradiation in the absence of photocatalyst still triggered the formation of HQ, and CT, but to a much lower extent. This indicates that photolysis of C–H and/or C−Cl bonds on the aromatic ring and possibly the incorporation of oxygen may have also led to oxidations to some extent. Additionally, several findings can be obtained from Figure 7. In the first phase, chlorohydroxyphenol was formed directly from DCP (oxidative dechlorination), and in the later phase it might have been also formed by hydroxylation of monochlorophenol itself, which had been previously generated by reductive dechlorination of DCP. The following pathways could be proposed: DCP ⇉ dichlorohydroxyphenols + PHN + chlorohydroxyphenol + [monochlorophenol ⇉ chlorohydroxyphenol].

*Sequential method.* In comparison, the decomposition pattern of PHN and DCP by SQ might have been similar to PC’s, except the concentrations of aromatic TPs were lower, as these are rapidly formed and destructed during OZ. Production of acids was also lower due to long PC (Figure 6), but SQ of PCP is an exception since most of it had been already effectively degraded by preozonation, and thus, acids’ production was higher. In the case of PHN (Figure 5e), HQ and CT were formed immediately with OZ, and during PC their concentrations remained mostly unchanged. Moreover, in the case of PCP (Figure 5f), with previous OZ, it was possible to lower the TH evolution extent effectively, which would have otherwise been formed by PC.

**Figure 8:**
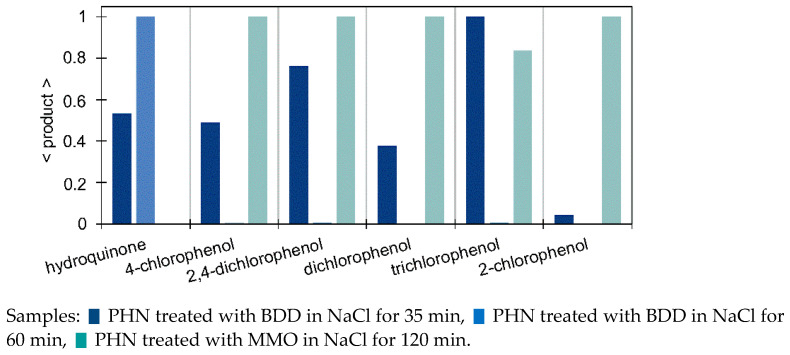
Relative amount of aromatic products (see Equation (4) in Section 2.4) in electrooxidized samples PHN determined by SPME/GC–MS (normalized for each product to the highest peak area of the three analyzed samples).

*Electrooxidation.* In the case of BDD/NaCl, 2,4-dichlorophenol was detected up to concentration 29.0 mg/L; in addition, approximately six times higher concentrations were determined after 160 min of MMO/NaCl than after 35 min of EO with BDD/NaCl. A similar trend can be concluded for other chlorophenols (Figure 8). During EO/Na_2_SO_4_, HQ was detected in all cases in concentrations < 0.5 mg/L, whereas lower concentrations of CT were found only in the BDD/160 min sample. BQ evolved in all samples, regardless of the anode and electrolyte used. In addition, degraded PHN was almost entirely converted into BQ after 160 min of MMO/Na_2_SO_4_ treatment. Acid formation was relatively low, which is proportional to a slow degradation process (Figure 6).

### 3.4. Detoxification Estimated from Acute Ecotoxicity Tests with Daphnia magna

Detoxification of pollutants is the ultimate goal of all degradation technologies, and not just the removal of parent phenols. Therefore, tests on aquatic invertebrates, such as water fleas, are extremely important, as these organisms frequently come into contact with pollutants, which can affect the whole freshwater ecosystem. The advantage of the applied tests is the ability to non-specifically evaluate the inhibitory and biological effects of mixtures of all known and also unknown TPs. Results are shown in Table 3, showing determined 48-h acute inhibition of the mobility of *Daphnia magna* (see Equation (6) in Section 2.4) after incubation of test organisms in diluted samples (AOP-treated phenols).

*Ozonation.* Rapid and complete dechlorination (see [21,24]), high preference to break down phenols’ aromatic ring and progressive formation of accumulative organic acids, seen as a sudden drop in pH [16,17,18,27], are the reasons for rapid detoxification (Table 3). OZ allowed for quick detoxification which was achieved after less than 1 min treatment, which is similar to findings in [26]. This is true for OZ of 50 and also 10 mg/L of chosen phenols (Table 3). Moreover, inhibition on *D. magna* did not increase during treatment, which means that less toxic TPs—relative to DCP and PCP—were formed (Table 3). However, this is not the case for PHN, where the first increase in inhibition might have been the result of the formation of oxidized aromatics (e.g., 48hEC_50_ ratio for HQ and 4,4′-biphenydiol relative to PHN is 0.004 and 0.1 for *D. magna*, respectively [46]), and the second increase perhaps due to oxalic acid formation [17].

*Photocatalysis and Photooxidation.* Described facts on PC in sections above are the reason for slow decline in inhibition of the treated samples, and even slower detoxification reached by PO (Table 3). Uniquely for PHN, an increase in inhibition was characteristic for both concentrations used. This was due to the formation of highly oxidized aromatics, such as (chloro)hydroxyphenols, CPs, and (polychlorinated) phenoxyphenols/biphenyls, which tend to be more toxic and bioavailable to *D. magna.* For example, 48hEC_50_ ratio for DCP, PCP, HQ, and triclosan relative to PHN is 0.1, 0.007, 0.004, and 0.02 for *D. magna*, respectively [46]. If transversely compared, inhibitions were higher and longer lasting (despite the same dilution factor) when higher initial concentrations were used (Table 3).

*Sequential method.* SQ provided immediate detoxification by ‘flash’ OZ, which otherwise could not be readily achieved by PC alone. Only in the case of PHN, again, inhibition increased and persisted (Table 3); most likely due to coupling products and photolysis reactions of TPs themselves.

*Electrooxidation.* During EO/NaCl, inhibition was significantly increased, e.g., MMO inhibition was 100% even after 120 min treatment (Table 3). This was most likely due to the presence of chlorophenols, which are much more toxic than PHN (e.g., 48hLC_50_ for 2-chlorophenol, 2,4-dichlorophenol, and 2,4,6-trichlorophenol relative to PHN is 0.35, 0.21, and 0.17, respectively, for *D. magna* [47]). Such a remarkable increase in toxicity represents an unfavourable decomposition process of PHN. On the other hand, despite the initial 94% inhibition, complete detoxification followed in the case of BDD after 35–60 min electrolysis (Table 3). This was due to the gradual decomposition of chlorinated aromatics into much less toxic and volatile chlorinated alkenes and carbonyl compounds, according to 48hLC_50_ data for *D. magna* in [47]. In the case of EO/Na_2_SO_4_, after 160 min of treatment with BDD, a final 0%*_inh_* could be achieved, but with MMO as much as 100%*_inh_* was measured (Table 3). Nevertheless, there was an intermediate increase in inhibition, which was possibly a result of the increased amount of BQ and other analogous aromatics. For example, in MMO, BQ was the predominant TP that accumulated. In conclusion, results discussed in the previous sections greatly reflect in ecotoxicological data, that can be also compared to tests on other organisms, e.g., *L. sativa* [17] and *V. fischeri* [44].

## 4. Conclusions

The study is focused on a representative family of ubiquitous and genotoxic pollutants: phenolic compounds, namely, phenol, 2,4-dichlorophenol and pentachlorophenol. Their chemical fate and potential impact on water organisms during four types of AOP treatments (ozonation, photocatalytic oxidation with immobilized *N*-TiO_2_ thin films, their sequence, and anodic electrooxidation) were investigated, using a variety of complementary techniques for instrumental analysis along with ecotoxicological assessment.

Results indicate that ozonation causes a favourable decomposition process from all viewpoints, which includes rapid target degradation (depending on structure, presence of co-substrates and initial concentration), fast and complete dechlorination, the rupture of aromatic structure and the progressive formation of organic acids. Incomplete mineralization but rapid detoxification is characteristic. Photocatalysis is a much slower degradation processes compared to ozonation. It shows the inability to open the aromatic ring efficiently and quickly, which reflects in the persistence of reactions on the aromatic ring and coupling of aromatics. The latter is manifested in increased inhibition of treated samples on *D. magna*, which only slowly decreases. An alternative approach for the destruction of pollutants might be seen in ozonation followed by photocatalysis, i.e., the sequential method, which is demonstrated to shorten the required time of degradation processes and to reinforce the dechlorination along with detoxification. As for electrochemical degradation, our results justify the use of more efficient BDD anode but also a need for proper electrolyte selection since the latter affect chemical transformations. Phenol removal is by far the fastest and most efficient in electrolyte NaCl, but it is accompanied by unfavourable formation of chlorinated transformation products. On the other hand, less effective degradation yet more favourable reactions and detoxification trends are provided by electrooxidation in Na_2_SO_4_.

Such a multidisciplinary approach to research the chemical degradation of pollutants induced by various AOPs is important for environmental protection. The use of many aspects of sample analysis with the combined expertise of analytical chemistry, environmental chemistry, environmental engineering, materials science, and ecotoxicology is rarely reported in the literature but is essential for evaluation of any AOP. In the future, in-depth research shall be performed in the field of (i) combination of complementary AOPs, such as ozonation followed by photocatalysis, in order to develop optimal sequential AOPs and to, therefore, minimize their limitations. Furthermore, (ii) there is also a necessity to assess phenols’ fate during treatment with AOPs in (semi)real matrices, suchlike model and real waste/surface waters. Finally, (iii) their natural chemical transformations and stability shall be investigated so as to model their real persistency. 

## Figures and Tables

**Table 1:** List of test mixtures in ultrapure water (MQ) containing 10 or 50 mg/L phenol (PHN), 2,4-dichlorophenol (DCP) or pentachlorophenol (PCP) treated with several AOPs, namely, ozonation (OZ), photocatalysis (PC), photooxidation (PO) ozonation followed by photocatalysis (sequential method, SQ), electrooxidation (EO) either with BDD or MMO anode in supporting electrolyte.

No.	AOP	Phenols	Approx. conc. (mg/L)	Solvent; Initial pH	Max. TT (min)
1	OZ	PHN	10	MQ; 8	60
		DCP	10		
		PCP	10		
2	OZ	PHN	50	MQ; 8	8
		DCP	50		
3	OZ	PHN, DCP, PCP	10, 10, 10 (mix)	MQ; 8	3
4	PC & PO	PHN	50	MQ, 8	180/300
		DCP	50		
		PHN	10		
		DCP	10		
		PCP	10		
5	SQ	PHN	20	MQ; 8	0.2 (OZ); 180 (PC)
		DCP	20		
		PCP	10		
6	EO/BDD EO/MMO	PHN PHN	50 50	2 g/L NaCl; 6	60 120
7	EO/BDD EO/MMO	PHN PHN	50 50	2 g/L Na_2_SO_4_; 6	160 160

**Table 2:**
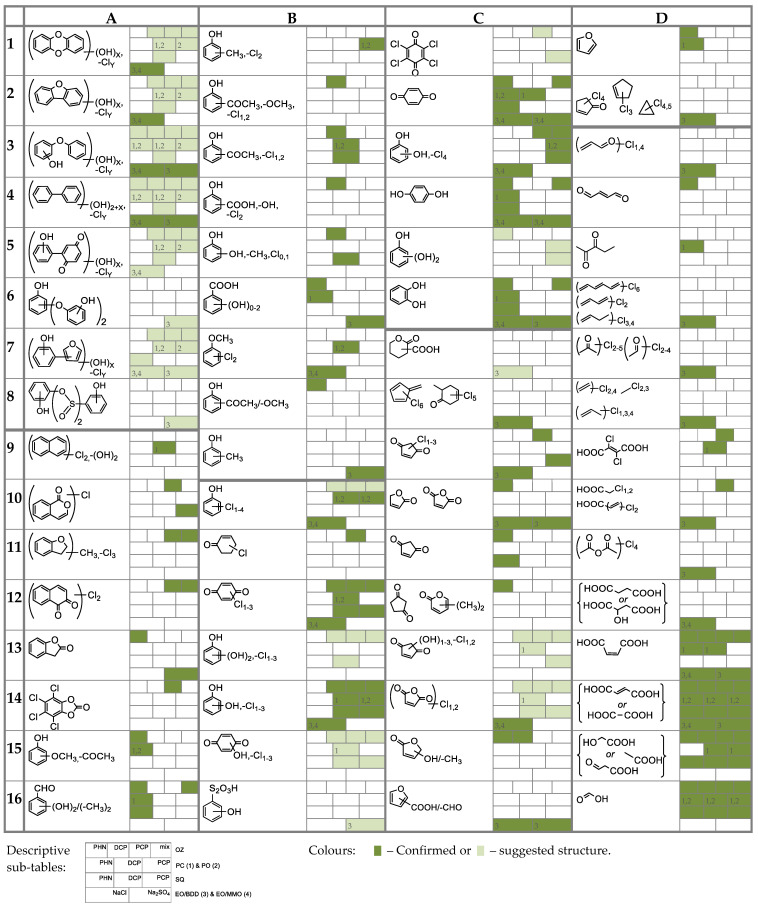
Transformation by-products (TPs) of phenol (PHN), 2,4-dichlorophenol (DCP), pentachlorophenol (PCP) and their mixture during ozonation (OZ), photocatalysis (PC; #1), photooxidation (PO; #2), sequential method (SQ), and electrooxidation (EO) with BDD (#3) and MMO (#4) anode in NaCl and Na_2_SO_4_ (for decription of table’s structure and colours, see footnotes).

**Table 3:** Comparison of the inhibition (%*_inh_*) on *D. magna* organisms (i.e., detoxification extent; see Equation (6) in Section 2.4) of treated phenol (PHN), 2,4-dichlorophenol (DCP), pentachlorophenol (PCP), and mixture solutions with ozonation (OZ), photocatalysis (PC), photooxidation (PO), sequential method (SQ) and electrooxidation with BDD and MMO in NaCl and N_2_SO_4_ at different treatment times (TT).

PHN	TT_0%_	TT_0–25%_	TT_25–50%_	TT_50–100%_	>TT_100%_
OZ (50 mg/L)					
OZ (10 mg/L)					
PC (50 mg/L)					
PC (10 mg/L)					
PO					
SQ		*^OZ^*	*^PC^*	*^PC^*	*^PC^*
BDD/NaCl					
MMO/NaCl					
BDD/Na_2_SO_4_					
MMO/Na_2_SO_4_					
**DCP**	**TT_0%_**	**TT_0–25%_**	**TT_25–50%_**	**TT_50–100%_**	**>TT_>100%_**
OZ (50 mg/L)					
OZ (10 mg/L)					
PC (50 mg/L)					
PC (10 mg/L)					
PO					
SQ		*^OZ^*	*^OZ^*	*^OZ & PC^*	*^PC^*
**PCP**	**TT_0%_**	**TT_0–25%_**	**TT_25–50%_**	**TT_50–100%_**	**>TT_>100%_**
OZ					
PC					
PO					
SQ		*^OZ^*	*^OZ^*	*^OZ & PC^*	*^PC^*
**Mixture**	**TT_0%_**	**TT_0–25%_**	**TT_25–50%_**	**TT_50–100%_**	**>TT_>100%_**
OZ *					

Legend: ▮ < 20%*_inh_*, ▮ 20−39%*_inh_*, ▮ 40−59%*_inh_*, ▮ 60−79%*_inh_*, ▮ > 80%*_inh_*; ▮ – not determined. Note: Notations ‘*OZ*’ and ‘*PC*’ were added in SQ. * TT*_x_*_%_ for PHN degradation.

## Data Availability

The data presented in this study are available on request from the corresponding author.

## Supplementary Materials

The following supporting information can be downloaded at: https://www.mdpi.com/article/10.3390/molecules27061935/s1, Table S1: Process parameters used for ozonation; Figure S1: HPLC–DAD chromatograms of (a) ozonation of DCP (initial conc. ~50 mg/L) at TT 0 min, (b) at TT 1.5 min, and (c) at TT 4 min, as well as (d) IC chromatograms at TT 0.5 min, and (e) at TT 4 min; Table S2: Process parameters used for photocatalysis and photooxidation (reference [48]); Figure S2: HPLC–DAD chromatograms of photocatalytic treatment of (a) PHN (initial conc. ~50 mg/L) at TT 180 min, of (b) DCP (initial conc. ~50 mg/L) at TT 90 min, and of (c) PCP (initial conc. ~10 mg/L) at TT 180 min, as well as (d) IC chromatogram of photocatalytic treatment of DCP (initial conc. ~50 mg/L) at TT 180 min; Figure S3: HPLC–DAD chromatograms of photooxidation of (a) PHN (initial conc. ~50 mg/L) at TT 180 min, (b) DCP (initial conc. ~50 mg/L) at 180 min, and of (c) PCP (initial conc. ~10 mg/L) at TT 180 min, as well as (d) IC chromatogram of photooxidation of DCP (initial conc. ~50 mg/L) at TT 180 min; Figure S4: HPLC–DAD chromatograms of the sequential method of: (a) PHN after flash ozonation and (b) after 120 min of photocatalysis; (c) DCP (initial conc. ~20 mg/L) after flash ozonation and (d) after 120 min of photocatalysis; Table S3: Process parameters used for anodic electrooxidations; Figure S5: HPLC–DAD chromatograms of electrooxidation of PHN (initial conc. ~50 mg/L) (a) by BDD in Na_2_SO_4_ at TT 160 min, (b) by MMO in Na_2_SO_4_ at TT 160 min, (c) by BDD in NaCl at TT 35 min, (d) by BDD in NaCl at TT 60 min, and (e) by MMO in NaCl at TT 120 min; Figure S6: General procedure of ecotoxicity tests following OECD Guidelines No. 202.

## Abbreviations

AOP (advanced oxidation process), BDD (boron-doped diamond), BQ (*p*-benzoquinone), CP (chlorophenol), CT (catechol), DCP (2,4-dichlorophenol), EO (electrooxidation), FM (fumaric acid), FO (formic acid), HQ (hydroquinone), MMO (mixed-metal oxide), MQ (ultrapure water), OX (oxalic acid), OZ (ozonation), PCP (pentachlorophenol), PHN (phenol), PO (photooxidation), SQ (sequential method, i.e., ozonation followed by photocatalysis), RD (relative descriptor calculated by Equation (5) in Section 2.4), TH (tetrachlorohydroquinone), TOC (total organic carbon), TP (transformation by-product), TT and TT*_x_*_%_ (treatment time and treatment time for reaching *x*% phenol removal).
