# Supplementary material for: Combined Analytical Study on Chemical Transformations and Detoxification of Model Phenolic Pollutants during Various Advanced Oxidation Treatment Processes
Source: Molecules. 2022 Mar 16;27(6):1935. doi: 10.3390/molecules27061935 (PMC8950015; doi:10.3390/molecules27061935)
Supplement: Supplementary file 1 [file molecules-27-01935-s001.zip › molecules-1600708-supplementary.pdf]

molecules

MDPI

Supplementary File

Combined analytical study on chemical transformations anddetoxification of model phenolic pollutants during variousadvanced oxidation treatment processes

1. Chemicals

Standards. Phenol (Sigma Aldrich, 99%), 2,4-dichlorophenol (Sigma Aldrich, 98%), pentachlorophenol (Sigma Aldrich, 99%), hydroquinone (Fluka, ≥ 99%), catechol (Fluka, ≥ 99%), tetrachloro-*p*-benzoquinone (Fluka, > 99%), oxalic acid (Fluka, ≥ 99%), maleic acid (Fluka, ≥ 99%), disodium fumarate (Fluka, 99%), formic acid (Honeywell, 98%), disodium succinate (Fluka, > 98%), acetic acid (Gram-Mol, > 99%), glycolic acid (Sigma Aldrich, 99%), glyoxylic acid monohydrate (Sigma Aldrich, 98%), malonic acid (Kemika, *puriss*), sodium chloride (Merck, *p.a.*). Tetrachlorohydroquinone was generated from tetrachloro-*p*-benzoquinone in 30% H_2O_2(aq)/MQ/acetone (0.1/1/8.9; *v/v/v*).

Reagents & solvents. Hydrogen peroxide (Sigma Aldrich, 30%, *aq*), sodium hydroxide (Sigma Aldrich, 50–52%, *aq*), sulphuric acid (Sigma Aldrich, 96%, *aq*), acetonitrile (Fisher Scientific, ≥ 99.9%), phosphoric acid (Sigma Aldrich, 85%, *aq*), ethyl acetate (Sigma Aldrich, ≥ 99.5%), *n*-hexane (Sigma Aldrich, ≥ 95%), ultrapure water (MQ; Millipore, USA).

Additional chemicals for identification of by-products: Lactic, gallic, citric acid, furane, resorcinol, *p*-benzoquinone, acetylfurane, pyrogallol, 2,5-dichlorophenol, glutaric acid anhydride, 4-hydroxybutanoic, 4-hydroxybenzoic, benzoic, 4-chlorobenzoic, acrylic, glutaric, adipic, trichloroacetic acid, 2,4-dichlorobenzoic, 3,5-dichlorobenzoic acid, 4-chlorobenzaldehyde. The listed chemicals had at least *p.a.* purity.

Materials used in AOPs. See sections 2–5.

2. Ozonation

Ozonation was conducted by continuous introduction of O_2/O_3 gaseous mixture (pregenerated from pure O_2 in ozonator MODULAR 8HC, Wedeco-Xylem) over a glass 300 mL batch reactor. O_2/O_3 mixture was introduced into mixture via a glass inlet, terminated with a porous frite. Experiments were conducted in highly ventilated chamber, at ambient temperature.

Table S1. Process parameters used for ozonation.

O_2/O_3 flow	15 L/h
pressure	0.5 bar
maximal O_3 production	15 mg/min
nominal O_3 concentration in gaseous mixture	60 g/m^3
initial sample volume	300 mL
mixing speed	600 m

2.1. Example chromatograms

Figure S1. HPLC-DAD chromatograms of **(a)** ozonation of DCP (initial conc. ~50 mg/L) at TT 0 min, **(b)** at TT 1.5 min, and **(c)** at TT 4 min, as well as **(d)** IC chromatograms at TT 0.5 min, and **(e)** at TT 4 min.

a)

b)

c)

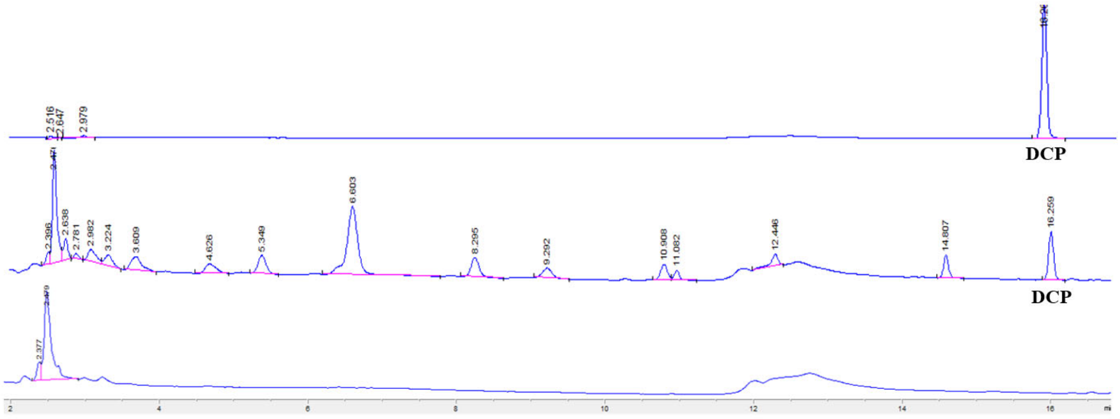

(d)

(e)

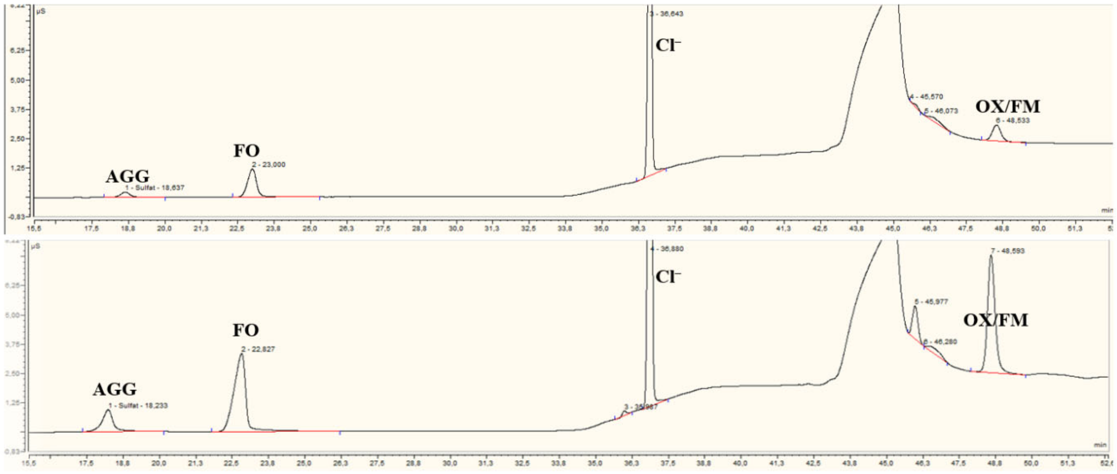

2.1. Photocatalysis and photooxidation

Photocatalytic treatment of the test solutions was performed in a static batch reactor (photocell) with immersed TiO_2^{im} glass plates on Teflon holder. A photocell with catalyst and test mixture was placed in UVA illuminator. Pure O_2 was continuously bubbled into the reactor mixture. In the case of photooxidations, processes were performed without TiO_2^{im} glass plates.

Table S2. Process parameters used for photocatalysis and photooxidation.

O_2 flow	25 mL/min
UVA illumination	6 light bulbs (UV Black Light Blue, 15W) emitting at λ_{max} 365 nm
catalysts	8 glass plates with immobilized *N*-doped TiO_2 ultrathin films
initial volume	90 mL
temperature	ambient (21–23 °C)

Synthesis of supported *N*-doped TiO_2 catalyst: In-detailed description is provided in ref. [1]. Synthesis involved inorganic sol-gel preparation of TiO_2 by mixing 3.68 mL of $TiCl_4$ (Fluka, > 98%) in 60 mL of distilled water with the addition of 4.95 mL of 12 M H_2SO_4 (Sigma Aldrich, 97%). Filtration of the clear dispersion was followed by the addition of 0.3% (*m*/*m*) hydroxypropyl cellulose (MW = 100.000 g/mol, 99%, Sigma Aldrich) and 2% (*m*/*m*) NH_4NO_3 (Zorka Šabac, > 99%) based on the amount of TiO_2. Thin layers of *N*-TiO_2 were applied on clean glass plates (dimensions: 1.5 cm × 7.5 cm), but prior to dipping into sol, plates were dipped into surfactant; 1% Etolat 60 (Teol) solution (dipping speed: 5 cm/min). Then, 3 layers of sol were coated with a dip-coater (dipping speed: 20 cm/min). After application of the first and second layers, the plates were dried at 300 °C for 15 min. After application of the third layer, calcination at 600 °C for 30 min followed.

2.3. Example chromatograms

Figure S2. HPLC-DAD chromatograms of photocatalytic treatment of **(a)** PHN (initial conc. ~50 mg/L) at TT 180 min, of **(b)** DCP (initial conc. ~50 mg/L) at TT 90 min, and of **(c)** PCP (initial conc. ~10 mg/L) at TT 180 min, as well as **(d)** IC chromatogram of photocatalytic treatment of DCP (initial conc. ~50 mg/L) at TT 180 min.

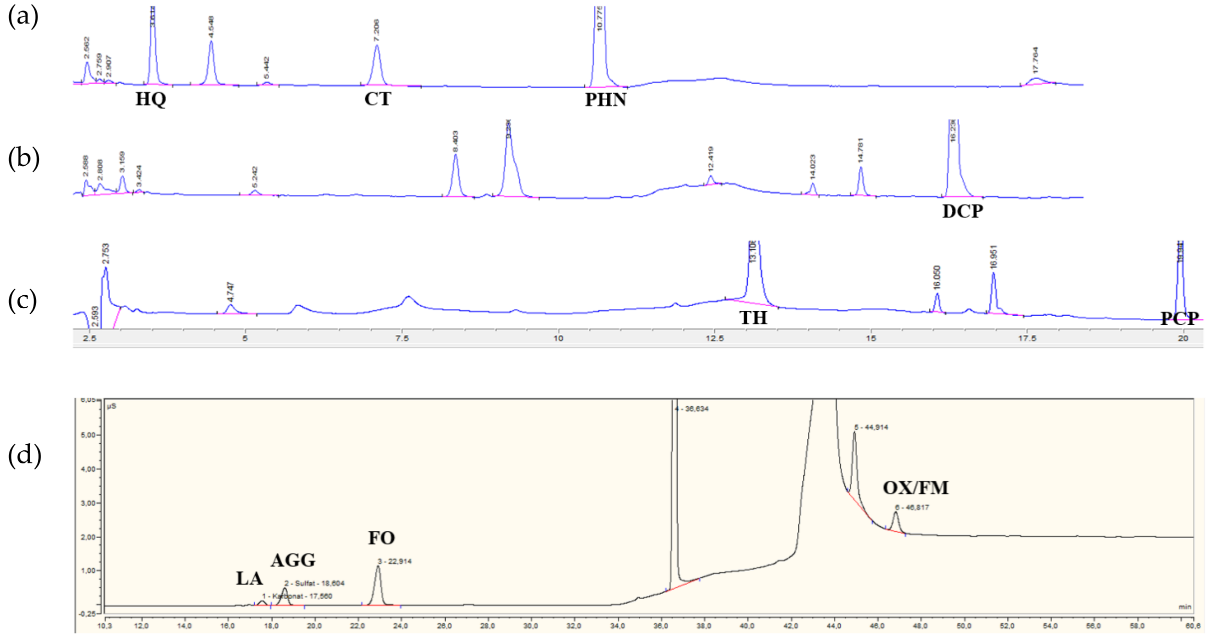

Figure S3. HPLC-DAD chromatograms of photooxidation of **(a)** PHN (initial conc. ~50 mg/L) at TT 180 min, **(b)** DCP (initial conc. ~50 mg/L) at 180 min, and of **(c)** PCP (initial conc. ~10 mg/L) at TT 180 min, as well as **(d)** IC chromatogram of photooxidation of DCP (initial conc. ~50 mg/L) at TT 180 min.

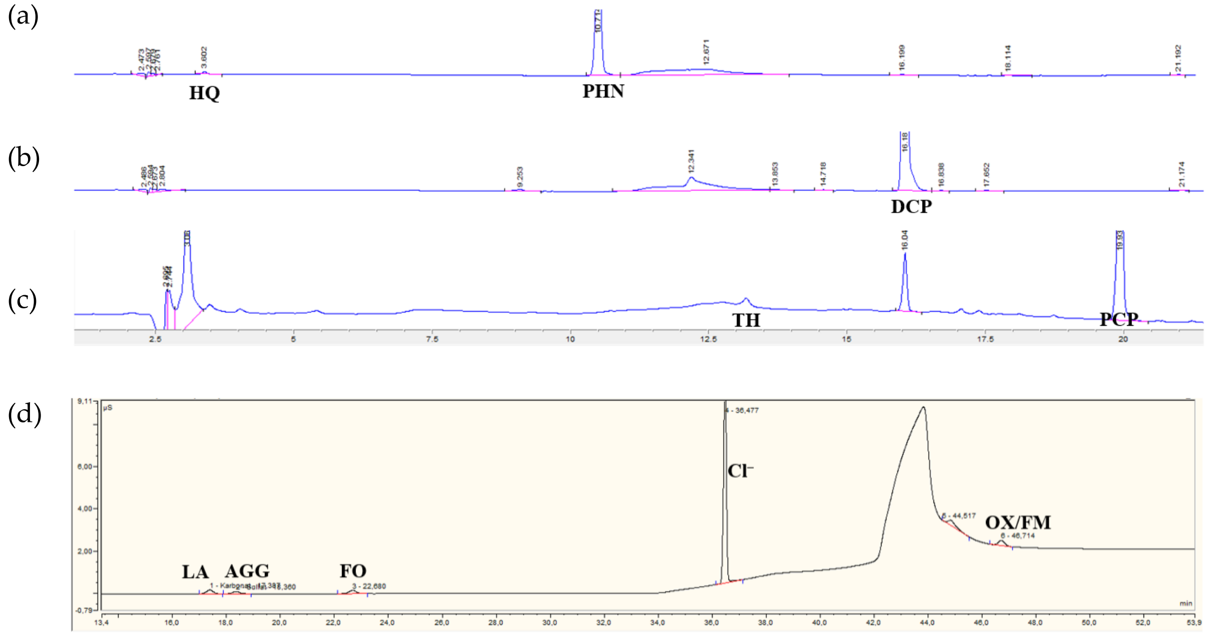

3. Sequential method

Process parameters were the same as for the individual ozonation or photocatalytic oxidation. After ozonation, ozonated sample were treated by photocatalysis.

3.1. *Example chromatograms*

Figure S4. HPLC-DAD chromatograms of sequential method of: **(a)** PHN after flash ozonation and **(b)** after 120 min of photocatalysis; **(c)** DCP (initial conc. ~20 mg/L) after flash ozonation and **(d)** after 120 min of photocatalysis.

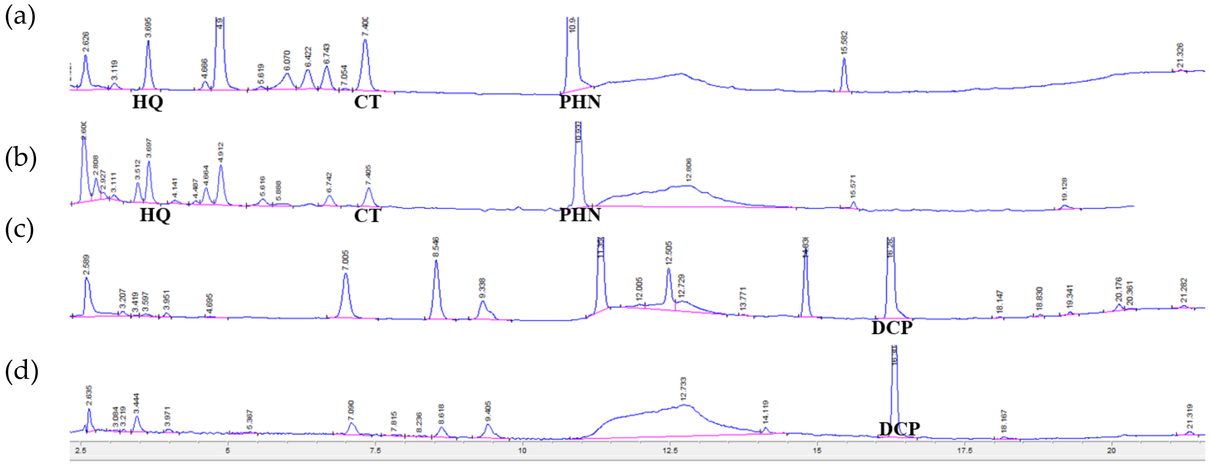

4. Electrooxidation

Prior to the treatment, electrodes were cleaned with diluted aqueous HCl (LachNer, 37%, *aq*) and then thoroughly washed with distilled water. The cathode was furtherly degreased with acetone (LachNer) and again washed with distilled water. After cleaning, test mixture (approx. 50 mg/L PHN in electrolyte) was transferred into the electrochemical cell (cathode *vs.* anode). Electrolysis for a certain time span was conducted in galvanostatic mode at ambient temperature. Chosen supporting electrolytes were used in different concentrations, but approximately the same conductivity was achieved (~3 mS/cm). The pH of the solutions containing phenol and electrolyte ranged from neutral to strongly acidic and was not additionally adjusted.

Table S3. Process parameters used for anodic electrooxidations.

current density	constant 20 mA/cm^2
distance anode/cathode	2 cm
power source	Atten, APS3005SI; 30V, 5A
anodes (material, shape)	(i) BDD (boron-doped diamond on Nb substrate, 28.26 cm^2 effective surface, mesh circular shape; Metachem, Germany),<br>(ii) MMO (mixed metal oxide RuO_2/IrO_2 on Ti substrate, 28.26 cm^2 effective surface, mesh circular shape; Metachem, Germany)
cathode	stainless steel (EN 1.4301/AISI 304; ≤ 0.07% C; 18.1% Cr; 8.2% Ni)
supporting electrolyte	2 g/L NaCl (LachNer, 99.5%); 2 g/L Na_2SO_4 (LachNer, 99%)
initial volume	400 mL
mixing speed	300 tations per minute

4.1. *Example chromatograms*

Figure S5. HPLC-DAD chromatograms of electrooxidation of PHN (initial conc. ~50 mg/L) **(a)** by BDD in Na_2SO_4 at TT 160 min, **(b)** by MMO in Na_2SO_4 at TT 160 min, **(c)** by BDD in NaCl at TT 35 min, **(d)** by BDD in NaCl at TT 60 min, and **(e)** by MMO in NaCl at TT 120 min.

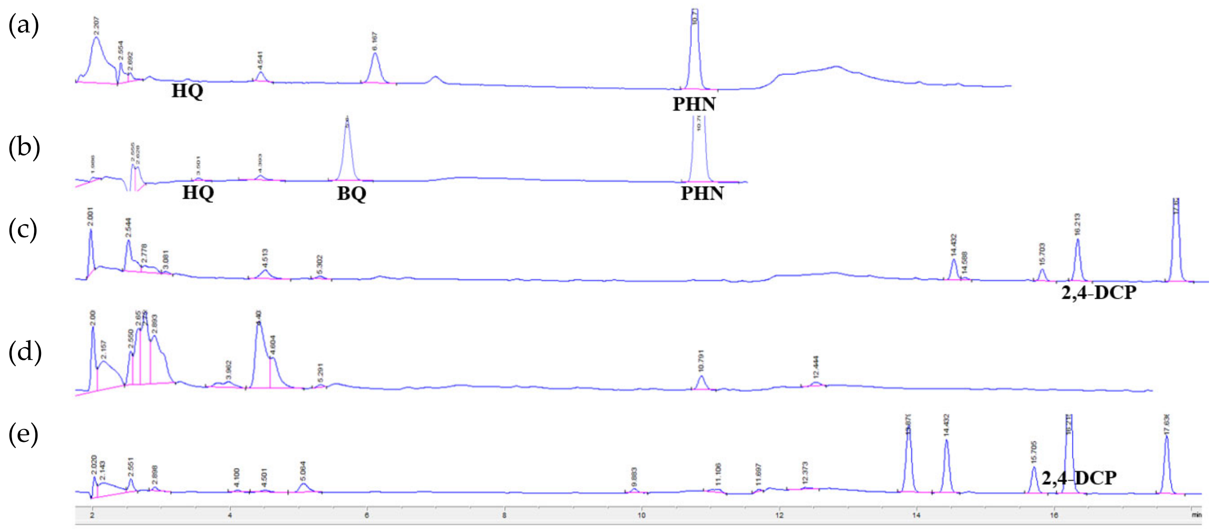

5. Ecotoxicity tests on *Daphnia magna*

Figure S6. General procedure of ecotoxicity tests following OECD Guidelines No. 202.

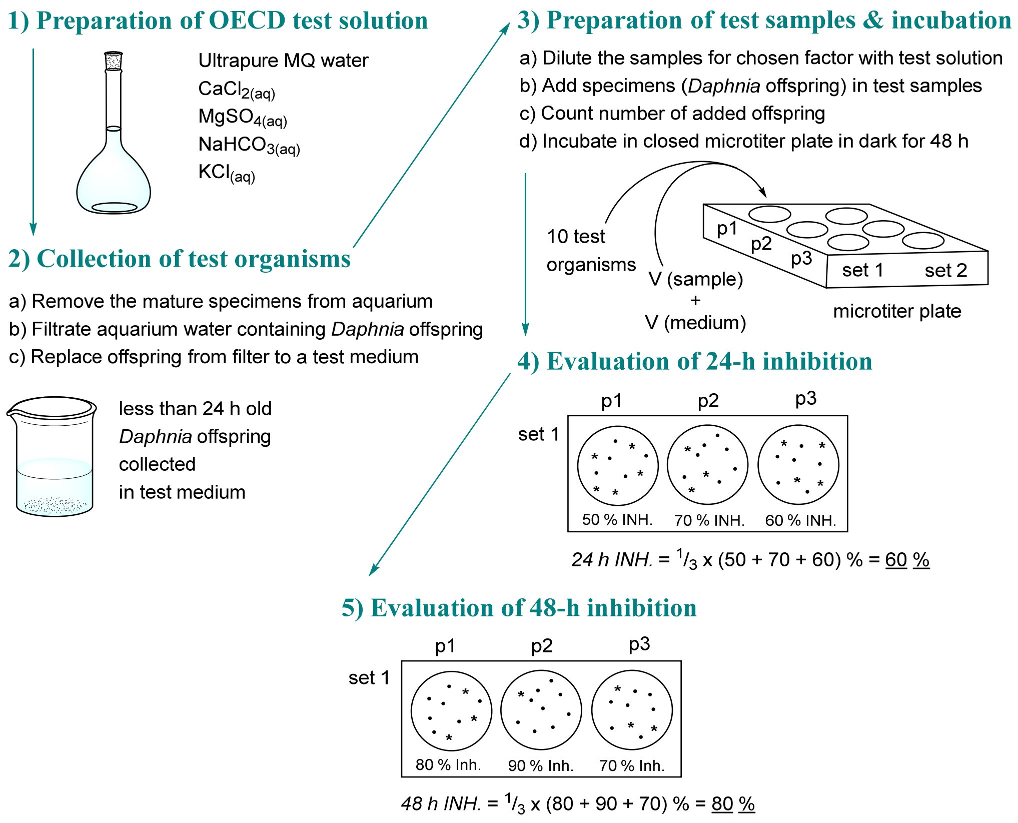

Testing of the individual samples was performed in 3 parallel assays, each containing 10 less than 24 h old *Daphnia* offspring. Specimens were placed in diluted test mixtures of the treated phenols at different treatment time (pH not previously adjusted). Laboratory microtiter plates with test samples were left at room temperature and in the dark for further 48 h. Organisms were not fed during the tests (48 h incubation). The final result of inhibition assays (expressed as $\%_{inh}$) was calculated as a ratio between No. of immobile organisms after 48 h of incubation in diluted samples and No. of all mobile organisms in

the diluted samples at the beginning of the test. For more details see Figure S6 or OECD Guidelines No. 202 [2].

References

1. Žener, B.; Matoh, L.; Carraro, G.; Miljević, B.; Korošec, R.C. Sulfur-, nitrogen- and platinum-doped titania thin films with high catalytic efficiency under visible-light illumination. *Beilstein J. Nanotechnol.* **2018**, *9*, 1629–1640, doi:10.3762/bjnano.9.155.
2. *OECD (2004), Test No. 202: Daphnia sp. Acute Immobilisation Test. In OECD Guidelines for the Testing of Chemicals, Section 2, OECD Publishing: Paris; pp. 1–12; https://doi.org/10.1787/9789264069947-en.*

Important abbreviations:

PHN (phenol)
DCP or 2,4-DCP (2,4-dichlorophenol)
PCP (pentachlorophenol)
TH (tetrachlorohydroquinone)
HQ (hydroquinone)
CT (catechol)
AGG (acetic and/or glycolic and/or glyoxylic acid)
FO (formic acid)
OX/FM (oxalic and/or fumaric acid)
BQ (*p*-benzoquinone)
TT (treatment time)
INH (inhibition on *D. magna*)
MMO (mixed-metal oxide)
BDD (boron-doped diamond)
IC (ion chromatography)
